# A botanical from the antiproliferative Cameroonian spice, *Imperata cylindrica* is safe at lower doses, as demonstrated by oral acute and sub-chronic toxicity screenings

**DOI:** 10.1186/s12906-020-03064-6

**Published:** 2020-09-10

**Authors:** Paul Nayim, Armelle T. Mbaveng, Arsene M. Ntyam, Victor Kuete

**Affiliations:** Department of Biochemistry, Faculty of Science, University of Dschang, Dschang, Cameroon

**Keywords:** *Imperata cylindrica*, Root methanol extract, Acute toxicity, Sub-chronic toxicity

## Abstract

**Background:**

The cytotoxicity of the root’s methanol extract of *Imperata cylindrica* (ICR). was previously reported in a panel of human cancer cell lines, including multi-drug resistant phenotypes. The aim of this study was to assess the acute and sub-chronic oral toxicity of methanol root extract of *Imperata cylindrica*.

**Methods:**

The acute toxicity was carried out according to the experimental protocol of OECD. The plant extract was administered orally to female rats at a single dose of 5000 mg/kg for 14 days and the animals were observed for any behavioral changes or mortality. For sub-chronic toxicity study, ICR was orally administered daily to male and female rats at different doses (250, 500 and 1000 mg/kg per b.w.) for 30 days. During these treatment days the animals were observed for any appearance of toxicity symptoms; following the treatment period, animals were sacrificed for hematological, biochemical and histopathology analysis.

**Results:**

From the results of the acute oral toxicity assay, ICR was found to be non-toxic at the dose of 5000 mg/kg b.w. During the period of sub-chronic toxicity test, observation of signs, behavior and health status of the animals showed no abnormality in the groups of animals treated with ICR as compared to the controls. Significant variation of the relative body weights of heart and kidney were observed at dose a 1000 mg/kg b.w. Significant decrease of aspartate aminotransferase, creatinine level, low density lipoprotein concentration, triglyceride and total cholesterol were observed. In males, we noticed a significant decrease of the level of granulocytes with an increase of lymphocytes and mean corpuscular hemoglobin concentration levels. Histological examinations performed on kidney and liver showed a normal kidney architecture and liver also presented a normal hepatic architecture with slight degeneration at a dose 1000 mg/kg b.w.

**Conclusion:**

ICR is safe for acute oral administration; however, for long-term oral administration, safety measures should be taken. Thus, oral sub-chronic exposure of ICR at lower doses are recommended while higher doses around 1000 mg/kg b.w. should be discouraged.

## Background

The fight against many human diseases mainly requires research and discovery of therapeutic substances; the latter may be derived from the animal kingdom as well as the plant kingdom. Plants synthesize certain class of chemical substances during their growth to resist against environmental stress, and some of those substances are used to cure human and animal diseases [1–3]. The rhizomes of *I. cylindrica* have been used as diuretic, anti-inflammatory or antipyretic agent in traditional herbal medicine [4]. Also, numerous studies had already shown biological effects of several medicinal plants towards human and animal diseases, such as *Imperata cylindrica* for its antibacterial [5], antifungal [6], anti-trypanosome [7] and anti-cancer activity. The cytotoxicity of ICR was previously reported in a panel of human cancer cell lines, including multi-drug resistant phenotypes; the methanol extract of this plant has been shown to induce apoptosis in leukemia CCRF-CEM cells, mediated by the alteration of mitochondrial membrane potential [8]. According to in vitro studies done on the leaf extract of *Imperata cylindrica*, this plant affects the proliferation of oral squamous cell carcinoma cell line SCC-9 through apoptosis induction [9]; also, investigations of Kwok et al. showed the cytotoxic and pro-oxidative effects of *Imperata cylindrica* aerial part ethyl acetate extract in colorectal cancer [10]. The methanol extract of the roots has been reported for its fascinating cytotoxic effects towards a panel of human cancer cell lines, including multidrug resistant phenotypes [11]. These cell lines included CCRF-CEM, CEM/ADR5000, HL60 and HL60AR leukemia cell lines, MDA-MB231 and MDA-MB231/*BCRP* breast adenocarcinoma cell line, HCT116 *p53*^+/+^ and HCT116 *p53*^−/−^ colon carcinoma cell line, U87MG, and U87MG.*ΔEGFR* gliobastoma cell line, HepG2 hepatocarcinoma cell line and Mia Paca2 pancreatic cancer cell line [8, 10]. Many compounds with biological properties had been isolated from the rhizomes of *I. cylindrica* such as arundoin, cylindrin, fernenol, cylindol, cylindrene, graminones and imperanene [12–14]. Despite their pharmacological properties, plants can also be harmful for human health. In addition, some chemical or secondary metabolites produced by plant are toxins like substances, they may cause problems to humans or animals. They have both useful and harmful effects in human beings and animals [15]. They act in animal or human body using varying specific mechanisms involving receptors, transporters, enzymes and even genetic materials at specific cells and tissues [16]. The poisonous part of the plant can be the seed, root, leaf, stalk, fruit or the whole plant whereby even a relatively small amount either taken or administered can be harmful to the human body; however, the doses of these substances are the most important factor [15]. So, despite the biological activity exhibited by certain plants, the study of their toxicity remains particularly important. The present study was devoted to evaluating the acute and sub-chronic oral toxicity of the root’s methanol extract of *Imperata cylindrica* on experimental animals.

## Methods

### Animals

We used young healthy males and females *albino Wistar* rats, nulliparous and non-pregnant, aged between 8 to 12 weeks maximum, we obtained these animals at the Animal house of the Biochemistry Department of the University of Dschang. These rats were randomly selected and kept in their cages five days before the onset of the tests for their acclimation to the laboratory conditions. The temperature of the experimental room was maintained at 22 °C (±3 °C), artificial lighting was provided, alternating sequences of 12 h of darkness; the animals were housed in individual cages and fed with standard rat diet ad libitum and were allowed free access to water. The experimental protocols used for this study were approved by the Local Ethical Committee of the Faculty of Science (University of Dschang – Cameroon) and were designed in concordance with the internationally accepted standard ethical guidelines for laboratory animals use and care as described in the guidelines of the European Union Institutional Ethics Committee on Animal Care (Council EEC 86/609/EEC of the 24th November 1986). All sections of this report adhere to the ARRIVE Guidelines for reporting animal research [17] (Additional file 1). A completed ARRIVE guidelines checklist is included in Checklist S1.

### Plant material and extract preparation

The roots of the plant used in this study were harvested at a field the West-Cameroon Region of Cameroon (“Menoua” Division) after the approval of the research project by the University of Dschang (Faculty of Science). A sample of this plant has been identified and authenticated by Mr. NANA Victor at the National Herbarium of Cameroon (Yaounde) as *Imperata cylindrica* (L.) Raeusch (Gramineae) under the voucher number 30139/SRF-Cam.

The roots of *Imperata cylindrica* were dried and ground, the resulting powder was macerated using methanol (1,3 w/v) at room temperature for 48 h. The mixture was stirred about 3 to 4 times per day to maximize yield. It was then filtered with Wattman N°1 paper and the filtrate obtained was evaporated using a rotary evaporator (BÜCHI R - 200) at 65 °C. The crude extract was recovered in a sterile vial and dried in an oven at 40 °C until the solvent completely evaporated.

### Acute oral toxicity study

Acute toxicity assessment of *Imperata cylindrica* was carried out according to the experimental protocol proposed by OECD Guideline 425 [18]. We performed the test at 5000 mg/kg b.w. of ICR. We had two experimental groups both made up of female rats: test group (*n* = 5) and control group (n = 5). The animals were kept under food depravation (removal of food, but not water) the day before the oral administration of the extract. After the fasting period, we weighed the rats and the plant extract was administered by gavage to animals at a single dose as mentioned above. Doses were calculated based on the fasting body weight of each animal. After administration of the extract, the rats were deprived from food for 4 h. The medium lethal dose (LD_50_) value was recorded 48 h after the extract was administered. The rats were observed individually with particular attention during the first 4 h after gavage and regularly during the first 24 h and then daily for 14 days during which physical symptoms of toxicity such as sensitivity to pain, motor activity by observing movements within the cage, noise sensitivity, tooth grinding and faeces appearance, tremors, drowsiness and convulsions. After these 14 days of observation, each animal was sacrificed to detect possible macroscopic pathological changes on organs such as the liver, kidneys, lungs and heart. The organs of the animal’s test group were compared to those of control group [18, 19].

### Sub-chronic oral toxicity study

In order to obtain information on repeated oral exposure to ICR, we assessed the sub-chronic toxicity of this plant according to the OECD Guideline No. 407 for chemical testing [20].

Nulliparous and non-pregnant *Wistar* rats aged between 8 to 12 weeks were divided into four groups of 10 rats per group, including 5 males and 5 females. Animals of the control group (group 1) received the vehicle daily (water + Tween 5%) while those of the remaining three test groups, that is group 2,3 and 4 received plant extract by oral gavage at doses 250, 500 and 1000 mg/kg b.w per day respectively. The animals were fed with standard rat diet ad libitum and were allowed free access to water. After the test period expired (30 days), we proceeded to animals sacrifice: the animals were anesthetized through intra-peritoneal injection of a cocktail containing ketamine (100 mg/Kg) and xylazine (12 mg/Kg) [ratio 10:1] for an 1 h.

### Collection of blood samples

After general anesthesia of animals, blood samples were collected: the animals were placed in supine position, the abdominal cavity opened, the intestines moved to the left, the abdominal aorta was located, 10 ml syringe needle was inserted at the base of the aorta and immediately the maximum amount of blood was collected and the animals death verified [21]. As soon as the blood sample was collected, it was separated in two parts; one part of the blood for haematological analyses, collected in tubes coated with ethylene diamine tetra-acetic acid (EDTA) and the other part of the blood was collected in tubes without anticoagulants and used for biochemical analyses. Blood samples for evaluation of biochemical parameters were kept at room temperature for 1 h to allow coagulation; they were then centrifuged at 300 rpm for 10 min. The serum obtained was introduced into eppendorf tubes and stored at − 25 °C for future use.

### Evaluation of haematological parameters

Blood samples collected in the EDTA tubes were used to perform blood count using an impedance hematology machine (QBC Auto-read Plus, United Kingdom). The hematological parameters analyzed included leukocytes, neutrophils, eosinophils, basophils, lymphocytes and monocytes counts; red blood cells (RBC), hemoglobin (Hb), mean corpuscular volume (MCV); mean corpuscular hemoglobin (MCH), mean corpuscular hemoglobin concentration (MCHC), platelets (PLT), Hematocrit (HCT), Mean corpuscular volume (MCV), Mean corpuscular Haemoglobin (MCH), Mean corpuscular Haemoglobin concentration (MCHC), Red blood cells distribution width CV, Red blood cells distribution width SD.

### Assessment of biochemical parameters

In this study, we explored the following biochemical parameters: aspartate aminotransferase (AST), alanine aminotransferase (ALT), proteins, urea, creatinine, total cholesterol (TC), high density lipoprotein (HDL), low density lipoprotein (LDL), triglyceride (TG). Handling was carried out according to the protocol of the kit manufacturer.

### Histology

After the animals were sacrificed, the heart, liver, lungs, spleen and kidneys were collected and weighed but only the liver and the kidney were used for histological analyses [22]. Collected organs were washed in a 0.9% NaCl solution and immediately preserved in 10% formaldehyde, dehydrated with alcohol; 5 μm thick cuts were made using a microtome and stained with hematoxylin eosin. These colored sections were covered with a thin glass plate and observed under an optical microscope equipped with a camera (400X) for taking photographs. A comparative histopathological study of the organs was performed.

### Statistical analysis

Data from each experimental group was expressed as mean *±* SEM. One-way analysis of variance (ANOVA) followed by Dunnett’s post-hoc test for multiple comparisons were used for statistical analysis of data using GraphPad Prism software version 5.01. Differences were considered significant at a probability level of 5% (*p* < 0.05).

## Results

### Acute toxicity study

Acute toxicity assessment of ICR was to determine the dose at which it kills the 50% of test animals (LD_50_) and the highest dose for which no toxic effects is observed in relation to the control lot. Forty-eight hours (48H) after administration of the single dose of 5000 mg/kg b.w. of ICR, we did not record any animal deaths neither during the 14 days of monitoring and observation. Immediately after the extract administration, some animals experienced agitation and drowsiness; however, these signs disappeared within few minutes. Thus, the LD_50_ of ICR was estimated to be greater than 5000 mg/kg b.w.

### Sub-chronic toxicity study

Throughout the test period (30 days), at the different extract doses, no visible sign of toxicity was observed in both the males and the females test and control groups.

### Effects of oral administration of ICR on body weights and relative organ weights

**Fig. 1:**
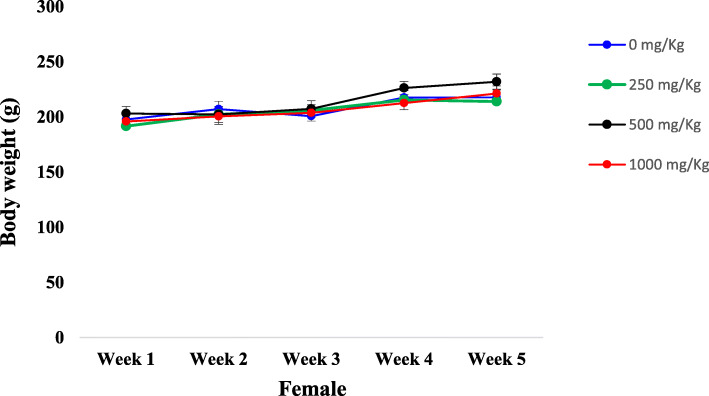
Initial and weekly body weight measurements (g) of the female rats after 30 days of sub-chronic toxicity study of ICR. Results were expressed as the mean S.E.M. of *n* = 5 rats. (Significantly different from the control; * *p* < 0.05)

**Fig. 2:**
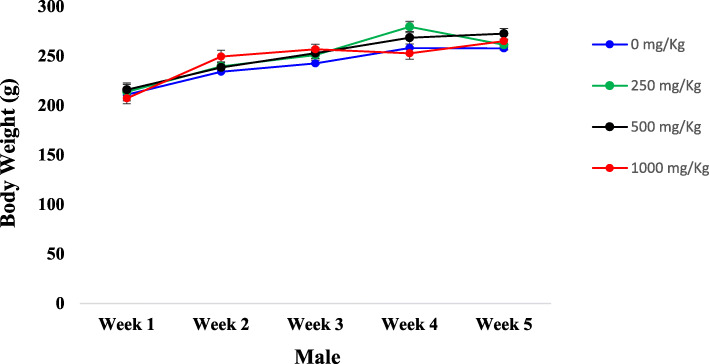
Initial and weekly body weight measurements (g) of the male rats after 30 days of sub-chronic toxicity study of the ICR. Results were expressed as the mean S.E.M. of n = 5 rats. (Significantly different from the control; * *p* < 0.05)

Figures 1 and 2 summarize the evolution of animal body weights both in females and males, respectively. Overall after the five (05) weeks of the test, we observed in all groups, an increase of rat body weights with a slight non-significant decrease of body weights both for male and female rats treated with 500 mg/kg b.w. in the last week of the test.

Tables 1 and 2 present the relative organ weights of the control group animals and those of rats treated with ICR at different doses (250, 500 and 1000 mg/kg b.w.) in females and males respectively. All females of the test groups showed a non-significant decrease (*p* < 0.05) of relative masses of organs such as the heart and spleen compared to the control group. In males, there was a significant increase (p < 0.05) of the relative weight of heart in rats treated with 1000 mg/kg b.w. compared to controls. We also noticed a significant increase (*p* < 0.05) of kidney relative weigh in test group rats at 500 and 1000 mg/kg b.w.

**Table 1:** Effects of sub-chronic oral administration of ICR on relative organs weights of females rats

Females	ICR (mg/kg b.w.)			
	Control	250	500	1000
**Liver**	3.442 **±** 0.348	3.510 **±** 0.350 ^ns^	3.562 **±** 0.167 ^ns^	3.638 **±** 0.247 ^ns^
**Heart**	0.333 **±** 0.017	0.291 **±** 0.001 ^ns^	0.314 **±** 0.017 ^ns^	0.328 **±** 0.025 ^ns^
**Lungs**	0.630 **±** 0.094	0.652 **±** 0.024 ^ns^	0.646 **±** 0.086 ^ns^	0.701 **±** 0.123 ^ns^
**Spleen**	0.405 **±** 0.103	0.364 **±** 0.024 ^ns^	0.362 **±** 0.023 ^ns^	0.365 **±** 0.003 ^ns^
**Kidney**	0.650 **±** 0.007	0.646 **±** 0.033 ^ns^	0.649 **±** 0.034 ^ns^	0.646 **±** 0.015 ^ns^

Results were expressed as Mean ± SEM for *n* = 5 animals per group. Significantly different from the control at *p* < 0.05; ns: non significative; ICR: methanol extract of the roots of *Imperata cylindrica*

**Table 2:** Effects of sub-chronic oral administration of ICR on relative organ weights of males rats

Males	ICR (mg/kg b.w.)			
	Control 2,505,001,000			
**Liver**	3.117 ± 0.224	3.244 ± 0.314 ^ns^	3.173 ± 0.138 ^ns^	3.485 ± 0.098 ^ns^
**Heart**	0.283 ± 0.008	0.293 ± 0.022 ^ns^	0.311 ± 0.013 ^ns^	0.333 ± 0.016*
**Lungs**	0.595 ± 0.056	0.650 ± 0.015 ^ns^	0.566 ± 0.030 ^ns^	0.656 ± 0.059 ^ns^
**Spleen**	0.355 ± 0.031	0.325 ± 0.010^ns^	0.392 ± 0.049 ^ns^	0.338 ± 0.013 ^ns^
**Kidney**	0.604 ± 0.003	0.618 ± 0.020 ^ns^	0.657 ± 0.020*	0.654 ± 0.023*

Results were expressed as Mean ± SEM for n = 5 animals per group. *:Significantly different from the control at *p* < 0.05; ns: non significative; ICR: methanol extract of the roots of *Imperata cylindrica*

### Effects of oral administration of ICR on biochemical parameters

Tables 3 and 4 present the results of biochemical parameters for animals of the control group and for those of animals treated at different doses of ICR (250, 500 and 1000 mg/kg b.w.) for females and males respectively. We noted a significant decrease (*p* < 0.05) of urinary creatinine concentration in both females and males treated with ICR at 500 mg/kg b.w.

**Table 3:** Effects of sub-chronic oral administration of ICR on serum and urinary biochemical parameters in females rats

Females	ICR (mg/kg b.w.)			
	Control	250	500	1000
**Serum creatinine (μmol/l)**	53.670 **±** 3.969	46.300 **±** 4.210 ^ns^	52.620 **±** 2.977 ^ns^	48.41 **±** 3.930 ^ns^
**Urinary creatinine (**μmol**/l)**	1049 **±** 67.97	1159 **±** 52.730^ns^	475.600 **±** 44.200*	994.900 **±** 55.250^ns^
**Serum urea (mmol/L)**	6.733 **±** 0.026	6.113 **±** 0.733^ns^	3.455 **±** 0.131^ns^	6.762 **±** 0.7427^ns^
**Urinary urea (mmol/L)**	10.739 **±** 0.024	11.46 **±** 1.260^ns^	12.100 **±** 0.5764^ns^	12.080 **±** 0.8529^ns^
**Serum total protein (g/L)**	44.800 **±** 0.034	43.300 **±** 0.062^ns^	34.500 **±** 0.056^ns^	42.300 **±** 0.007^ns^
**Urinary total protein (g/L)**	7.239 ± 0.277	6.800 ± 0.036 ^ns^	7.687 ± 0.241 ^ns^	6.606 ± 0.434 ^ns^
**ALT (U/L)**	33.350 **±** 1.521	31.650 **±** 2.260 ^ns^	35.200 **±** 1.218 ^ns^	31.830 **±** 1.701 ^ns^
**AST (U/L)**	74.740 **±** 3.420	58.220 **±** 2.019*	58.270 **±** 2.399*	52.100 **±** 2.895*

Results were expressed as Mean ± SEM for *n* = 5 animals per group. *:Significantly different from the control at *p* < 0.05

*ALT* Alanine aminotransferase, *AST* Aspartate aminotransferase, *ns* non significative, *ICR* methanol extract of the roots of *Imperata cylindrica*

**Table 4:** Effects of sub-chronic oral administration of ICR on serum and urinary biochemical parameters in males rats

Females	ICR (mg/kg b.w.)			
	Control	250	500	1000
**Serum creatinine (μmol/l)**	50.510 ± 1.500	46.300 ± 1.607 ^ns^	53.910 ± 1.730 ^ns^	51.560 ± 1.814 ^ns^
**Urinary creatinine (μmol/l)**	1088 ± 48.620	1079 ± 40.040^ns^	670.100 ± 30.000*	1091 ± 50.730^ns^
**Serum urea (mmol/L)**	5.650 ± 0.147	5.524 ± 0.175^ns^	5.697 ± 0.065^ns^	6.097 ± 0.336^ns^
**Urinary urea (mmol/L)**	25.180 ± 0.254	22.690 ± 0.221 ^ns^	24.490 ± 0.689 ^ns^	15.180 ± 0.261 ^ns^
**Serum total protein (g/L)**	45.300 ± 0.036	48.300 ± 0.044^ns^	44.900 ± 0.016^ns^	38.100 ± 0.026^ns^
**Urinary total protein (g/L)**	5.856 ± 0.059	6.271 ± 0.524 ^ns^	5.723 ± 0.193 ^ns^	5.395 ± 0.357 ^ns^
**ALT (U/L)**	34.630 ± 2.014	33.120 ± 2.365 ^ns^	32.00 ± 2.000 ^ns^	31.730 ± 2.026 ^ns^
**AST (U/L)**	68.770 ± 2.918	54.490 ± 2.258*	42.940 ± 2.029*	50.080 ± 1.747 *

Results were expressed as Mean ± SEM for *n* = 5 animals per group. *:Significantly different from the control at *p* < 0.05

*ALT* Alanine aminotransferase, *AST* Aspartate aminotransferase, *ns* non significative, *ICR* methanol extract of the roots of *Imperata cylindrica*

Whereas compared to control group, we also noted a significant decrease (p < 0.05) of the level of aspartate aminotransferase in all male and female rats treated with ICR at different test doses (250, 500 and 1000 mg/kg b.w.).

### Effects of oral administration of ICR on serum lipid profile

Tables 5 and 6 present the lipid profiles of the animals in the control group and those treated with ICR at different doses (250, 500 and 1000 mg/kg b.w.) in females and males, respectively. In females, compared to the control group, we did not notice any significant variation in lipid profile in animals treated with ICR; a similar observation was made in males’ rats. We observed a significant decrease of the concentration of low-density lipoprotein (LDL) in the group of animals treated with ICR at 250 mg/kg b.w. and a slight non-significant increase in total cholesterol (TC) and triglyceride (TG) levels.

**Table 5:** Effects of sub-chronic oral administration of ICR on the lipid profile of females rats

Females	ICR (mg/kg b.w.)			
	Control	250	500	1000
**TC (mmol/L)**	1.158 ± 0.173	1.699 ± 0.161 ^ns^	1.521 ± 0.172 ^ns^	1.337 ± 0.167 ^ns^
**TG (mmol/L)**	1.059 ± 0.141	0.843 ± 0.100 ^ns^	1.289 ± 0.175 ^ns^	0.994 ± 0.141 ^ns^
**HDL (mmol/L)**	0.946 ± 0.129	0.883 ± 0.072 ^ns^	0.809 ± 0.077 ^ns^	0.965 ± 0.070 ^ns^
**LDL (mmol/L)**	0.430 ± 0.023	0.442 ± 0.025^ns^	0.399 ± 0.0009 ^ns^	0.378 ± 0.045 ^ns^

Results were expressed as Mean ± SEM for n = 5 animals per group. *:Significantly different from the control at *p* < 0.05; TC: Total cholesterol; TG: Total triglyceride; HDL: High density lipoprotein; LDL: Low density lipoprotein; ns: non significative; ICR: methanol extract of the roots of *Imperata cylindrica*

**Table 6:** Effects of sub-chronic oral administration of ICR on the lipid profile of males rats

Males	ICR (mg/kg b.w.)			
	Control	250	500	1000
**TC (mmol/L)**	1.499 ± 0.100	1.699 ± 0.008 ^ns^	1.876 ± 0.062 ^ns^	1.378 ± 0.034 ^ns^
**TG (mmol/L)**	1.167 ± 0.072	1.21 ± 0.028^ns^	1.435 ± 0.221 ^ns^	1.248 ± 0.157 ^ns^
**HDL (mmol/L)**	2.193 ± 0.088	2.13 ± 0.052 ^ns^	2.425 ± 0.034^ns^	2.043 ± 0.199^ns^
**LDL (mmol/L)**	0.537 ± 0.110	0.343 ± 0.016*	0.5233 ± 0.023 ^ns^	0.645 ± 0.024 ^ns^

Results were expressed as Mean ± SEM for *n* = 5 animals per group. *:Significantly different from the control at *p* < 0.05

*TC* Total cholesterol, *TG* Total triglyceride, *HDL* High density lipoprotein, *LDL* Low density lipoprotein, *ns* non significative, *ICR* methanol extract of the roots of *Imperata cylindrica*

### Effects of oral administration of ICR on hematological parameters

Tables 7 and 8 present the hematological profiles of animals in the control group and those treated with ICR at different doses (250, 500 and 1000 mg/kg b.w.) in females and males, respectively. Males treated with ICR at 500 and 1000 mg/kg b.w. showed a significant increase (*p* < 0.05) in lymphocyte and MCHC levels; we also noted that females treated with the same doses of ICR showed a significant increase (*p* < 0.05) in PLT levels compared to the control groups. In all groups of females treated with ICR, there was a significant decrease (*p* < 0.05) in granulocyte levels; while in males, there was a significant increase (p < 0.05) in granulocyte levels compared to the control groups.

**Table 7:** Effects of sub-chronic oral administration of ICR on the hematological profile of females rats

Females	ICR (mg/kg b.w.)			
	Control	250	500	1000
**White blood cells (X 10**^**3**^ **/**μL**)**	18.650 **±** 0.450	15.3000 **±** 0.500 ^ns^	17.250 **±** 1.150 ^ns^	18.050 **±** 1.350^ns^
**Lymphocytes (%)**	82.850 **±** 1.750	84.700 **±** 1.825^ns^	80.570 **±** 0.602 ^ns^	77.500 **±** 0.900^ns^
**Granulocytes (%)**	14.870 **±** 1.365	10.550 **±** 1.150*	10.430 **±** 1.629*	10.700 **±** 0.900*
**Red blood cells (X 10**^**6**^ **/**μL**)**	7.590 **±** 0.130	7.160 **±** 0.150^ns^	7.340 **±** 0.100 ^ns^	7.000 **±** 0.040^ns^
**Haemoglobin (g/dL)**	16.300 **±** 0.360	16.130 **±** 0.585^ns^	16.280 **±** 1.415^ns^	15.750 **±** 0.550 ^ns^
**Hematocrit (%)**	44.200 **±** 2.000	41.130 **±** 2.573^ns^	41.600 **±** 3.677 ^ns^	41.300 **±** 1.500 ^ns^
**Mean corpuscular volume (fL)**	61.350 **±** 1.450	59.130 **±** 2.196^ns^	59.050 **±** 1.150 ^ns^	59.100 **±** 1.800^ns^
**Mean corpuscular Hb (pg)**	21.600 **±** 0.100	23.130 **±** 0.763^ns^	23.100 **±** 0.585 ^ns^	22.300 **±** 0.700^ns^
**MCHC (g/dL)**	37.200 **±** 1.100	38.300 **±** 1.200^ns^	38.800 **±** 1.20 ^ns^	38.050 **±** 0.050^ns^
**RDWCV (%)**	17.570 **±** 0.717	17.430 **±** 1.050^ns^	17.900 **±** 1.200^ns^	19.250 **±** 1.050^ns^
**RDWSD (fL)**	44.330 **±** 1.040	43.470 **±** 1.350 ^ns^	46.00 **±** 1.500 ^ns^	46.100 **±** 0.050 ^ns^
**Platelets (X 10**^**3**^ **/**μL**)**	521.00 **±** 8.900	512.0 **±** 9.800^ns^	824.300 **±** 6.506*	879.500 **±** 7.500*
**Mean platelet volume (fL)**	11.400 **±** 0.900	10.40 **±** 1.411^ns^	11.470 **±** 1.305^ns^	11.900 **±** 1.229^ns^
**Platelet distribution width (fL)**	11.770 **±** 1.002	10.330 **±** 1.193^ns^	10.200 **±** 0.900^ns^	11.770 **±** 1.002 ^ns^
**Plateletcrit (%)**	0.660 **±** 0.050	0.585 **±** 0.165^ns^	0.945 **±** 0.049^ns^	0.943 **±** 0.136^ns^

Results were expressed as Mean ± SEM for *n* = 5 animals per group. *:Significantly different from the control at *p* < 0.05

*MCHC* Mean corpuscular Haemoglobin concentration, *RDWCV* Red blood cells distribution width_CV, *RDWSD* Red blood cells distribution width_SD, *ns* non significative, *ICR* methanol extract of the roots of *Imperata cylindrica*

**Table 8:** Effects of sub-chronic oral administration of ICR on the hematological profile of males rats

Males	ICR (mg/kg b.w.)			
	Control	250	500	1000
**White blood cells (X 10**^**3**^ **/μL)**	18.300 ± 0.900	15.830 ± 0.419^ns^	18.530 ± 1.934^ns^	19.800 ± 1.400^ns^
**Lymphocytes (%)**	82.850 ± 5.250	73.170 ± 11.690^ns^	65.800 ± 1.900*	63.230 ± 3.150*
**Granulocytes (%)**	16.000 ± 2.384	23.000 ± 1.277*	23.300 ± 2.000*	24.350 ± 2.850*
**Red blood cells (X 10**^**6**^ **/μL)**	5.150 ± 0.694	5.483 ± 0.131 ^ns^	5.767 ± 0.754 ^ns^	5.187 ± 0.838 ^ns^
**Haemoglobin (g/dL)**	17.000 ± 1.200	15.930 ± 1.012^ns^	17.000 ± 0.500^ns^	14.630 ± 1.635^ns^
**Hematocrit (%)**	29.530 ± 1.351	29.100 ± 1.253 ^ns^	29.050 ± 1.650 ^ns^	27.590 ± 1.960 ^ns^
**Mean corpuscular volume (fL)**	82.530 ± 3.057	78.950 ± 4.122^ns^	81.650 ± 3.450^ns^	80.350 ± 4.450^ns^
**Mean corpuscular Hb (pg)**	34.170 ± 5.44	33.470 ± 6.130 ^ns^	32.000 ± 5.900 ^ns^	33.100 ± 4.700 ^ns^
**MCHC (g/dL)**	40.850 ± 2.550	40.600 ± 1.900 ^ns^	50.000 ± 2.300^*^	52.200 ± 2.010^*^
**RDWCV (%)**	18.000 ± 2.200	17.570 ± 2.203^ns^	19.070 ± 1.602^ns^	17.000 ± 1.400^ns^
**RDWSD (fL)**	44.370 ± 3.168	43.430 ± 3.350^ns^	65.250 ± 4.750 ^ns^	65.850 ± 4.050^ns^
**Platelets (X 10**^**3**^ **/μL)**	737.000 ± 60.000	600.0 ± 65.200 ^ns^	650.300 ± 58.800 ^ns^	651.300 ± 60.500 ^ns^
**Mean platelet volume (fL)**	11.650 ± 0.650	11.530 ± 0.650^ns^	11.400 ± 0.600^ns^	10.730 ± 0.611^ns^
**Platelet distribution width (fL)**	11.400 ± 0.800	11.000 ± 0.700^ns^	9.850 ± 1.150^ns^	10.200 ± 0.400^ns^
**Plateletcrit (%)**	0.840 ± 0.180	0.735 ± 0.205^ns^	0.870 ± 0.040^ns^	0.935 ± 0.165^ns^

Results were expressed as Mean ± SEM for n = 5 per group. *:Significantly different from the control at *p* < 0.05

*MCHC* Mean corpuscular Haemoglobin concentration, *RDWCV* Red blood cells distribution width_CV, *RDWSD* Red blood cells distribution width_SD, *ns* non significative, *ICR* methanol extract of the roots of *Imperata cylindrica*

### Histopathological examination

**Fig. 3:**
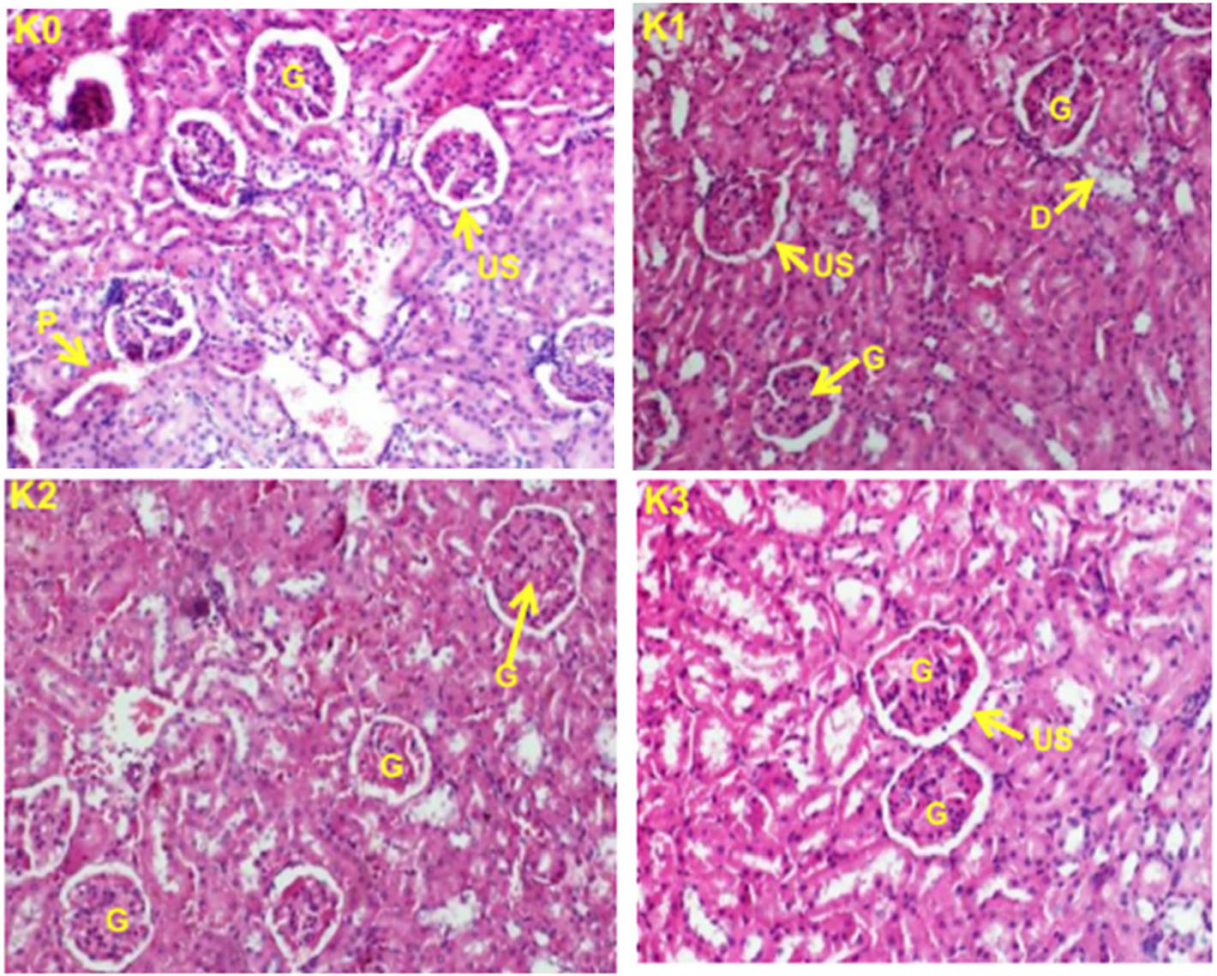
Kidney sections showing the effect of ICR after 30 days of sub-chronic toxicity study in females rats: (**K0**) Control group; (**K1**) 250 mg/kg; (**K2**) 500 mg/kg and (**K3**) 1000 mg/kg. Indicators: G: Glomerula, D: Distal tubule, P: Proximal tubule, US:Urinary space

**Fig. 4:**
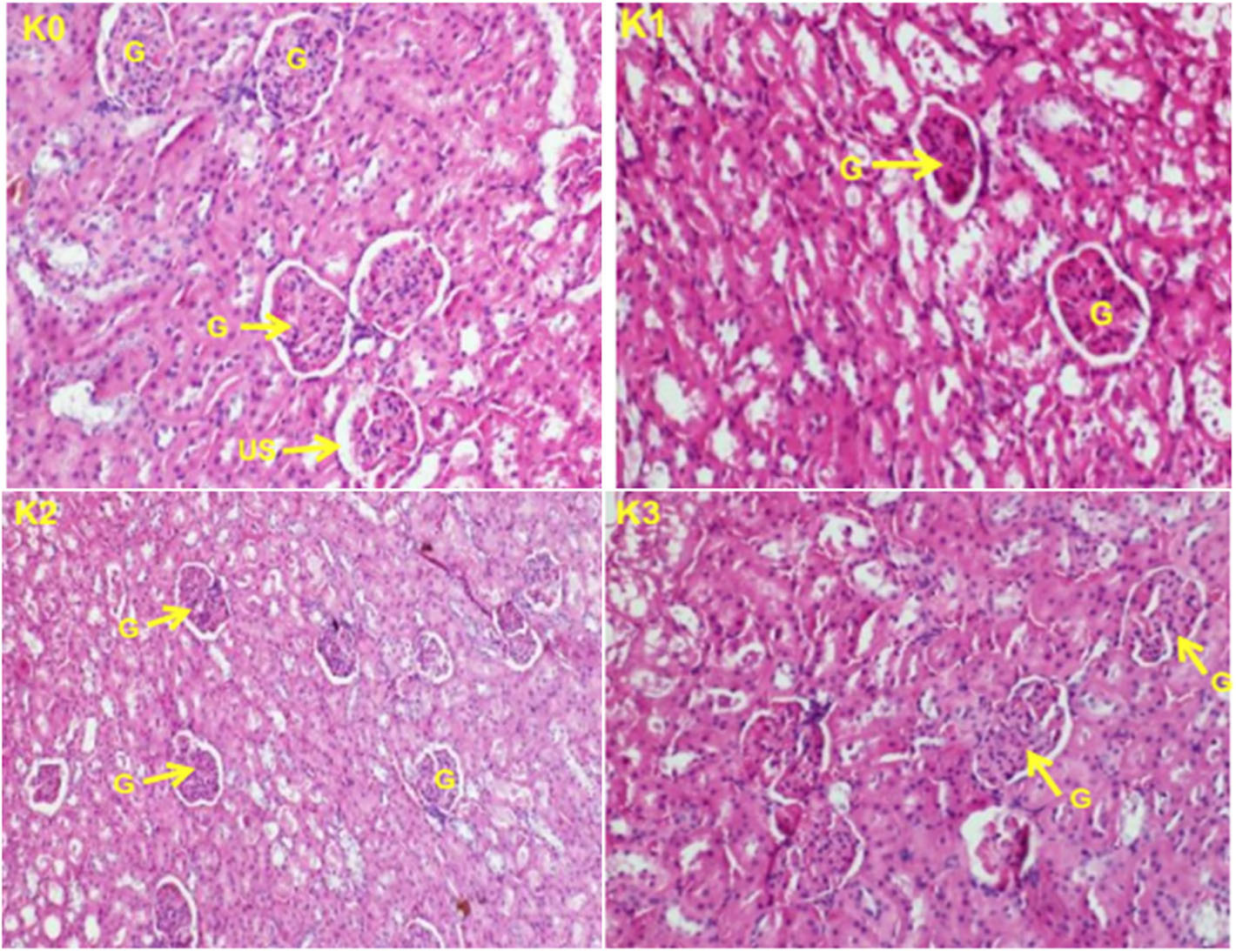
Kidney sections showing the effect of ICR after 30 days of sub-chronic toxicity study in males rats: (**K0**) Control group; (**K1**) 250 mg/kg; (**K2**) 500 mg/kg and (**K3**) 1000 mg/kg. Indicators: B: Glomelura and US: Urinary space

**Fig. 5:**
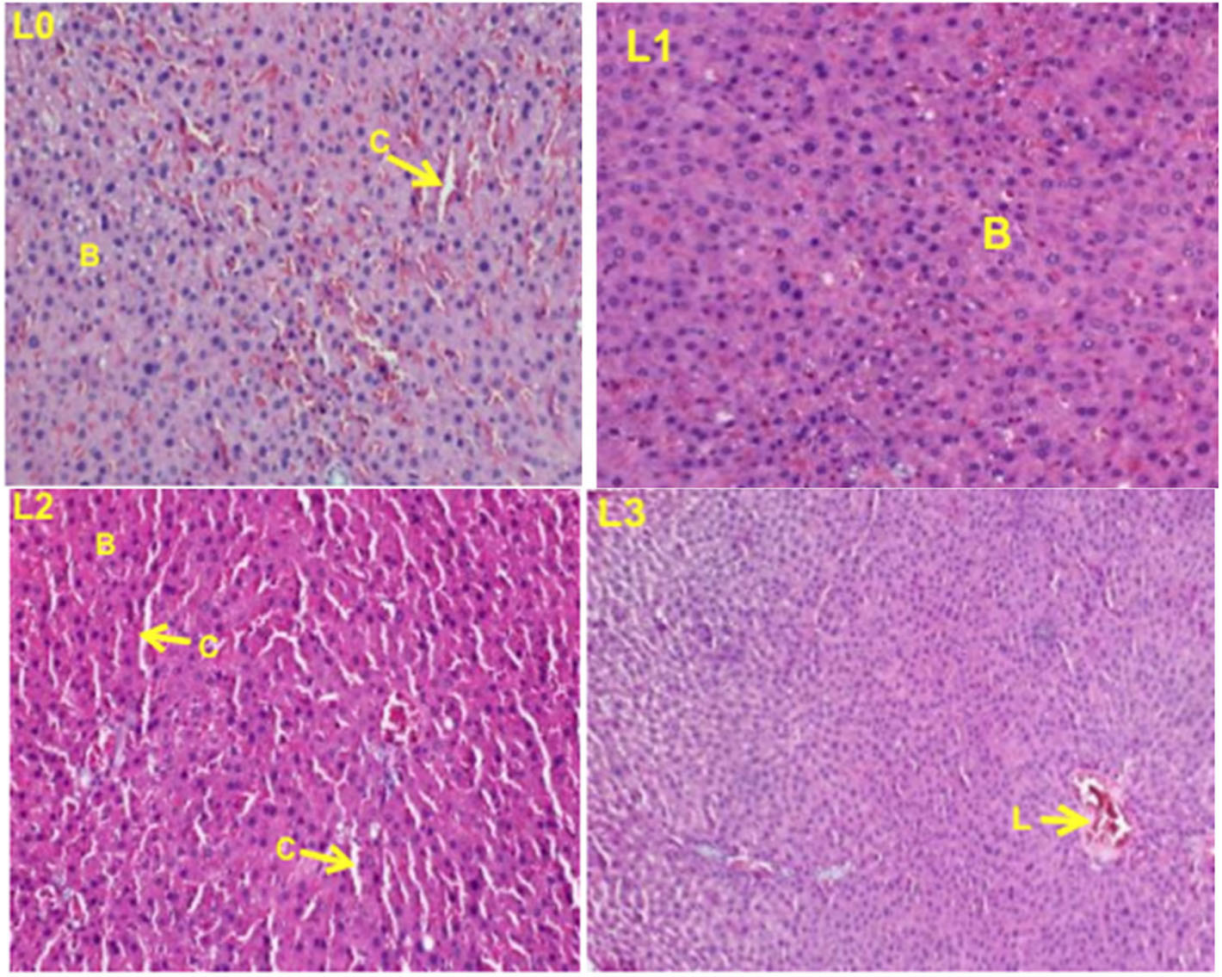
Liver sections showing the effect of ICR after 30 days of sub-chronic toxicity study in females rats: (**LO**) Control group; (**L1**) 250 mg/kg; (**L2**) 500 mg/kg and (**L3**) 1000 mg/kg. Indicators: B: Hepatocytes, C: Leukocytes infiltration, L: Cell lysis

**Fig. 6:**
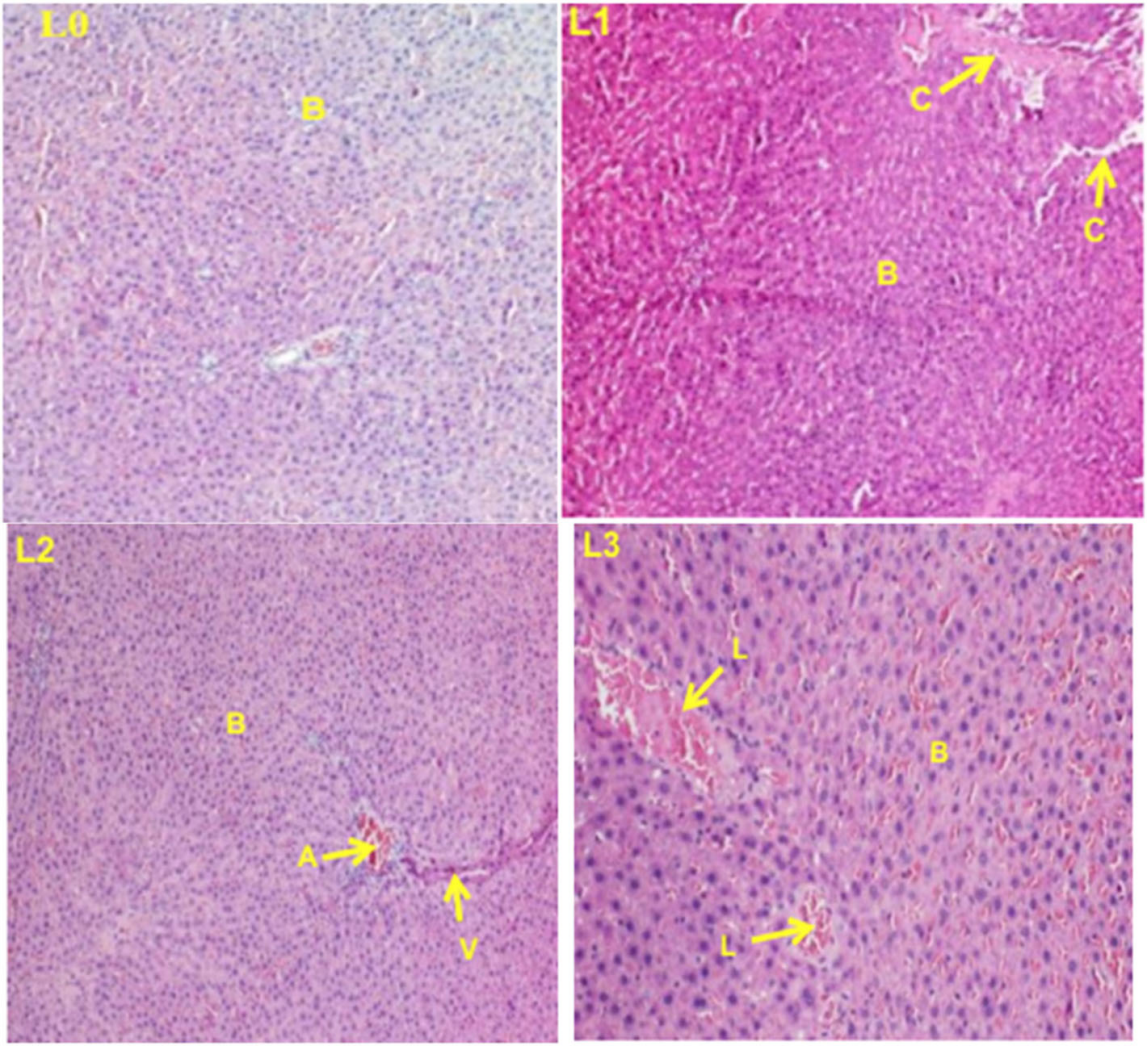
Liver sections showing the effect of ICR after 30 days of sub-chronic toxicity study in males rats: (**LO**) Control group; (**L1**) 250 mg/kg; (**L2**) 500 mg/kg and (**L3**) 1000 mg/kg. Indicators: B: Hepatocytes, C: Leukocyte infiltration, V: Centrilobular vein. L: Cell lysis

Histopathological examinations were performed on the liver and the kidney to assess whether organs or tissues had been damaged. The kidney of treated rats showed normal glomeruli and there was no necrosis of tubular epithelium in both the females (Fig. 3) and the males (Fig. 4) treated rats. The liver appeared normal with preserved hepatic architecture; however, slight degeneration (lesions) were occasionally observed in both the females (Fig. 5) and the males (Fig. 6) treated at the highest dose of ICR as compared to the control group.

## Discussion

The use of medicinal plants in the treatment of human diseases is not only assured by their identified biological properties, but also by the demonstration of their harmlessness to human cells. Numerous investigations on ICR have already highlighted its various pharmacological properties [5–10]. Several studies showed the anti-proliferative activity of various parts of ICR against a panel of human cancer cell lines, including multi-drug resistant phenotypes [8–10]. But up till date, no literature has reported toxicological studies on the methanol extract of the roots of this plant; hence our interest in undertaken the study of the acute and sub-chronic toxicity of ICR in order to contribute to its safe use in the management of certain human diseases.

In this study, acute oral toxicity was achieved by administering single dose of 5000 mg/Kg b.w. in females. At this dose, there were no lethality and no clinical signs of acute toxicity. The lethal dose 50 (LD_50_) of ICR would therefore be higher than 5000 mg/Kg b.w., and this result correlates investigations done by Chunlaratthanaphorn [23] on the aqueous extract of this same plant which also indicated an LD_50_ higher than 5000 mg/Kg b.w.

The sub-chronic toxicity of ICR was realized by repeated oral administration of 250, 500 and 1000 mg/Kg b.w. for 30 days.

After 30 days of treatment, weight gains were higher in the test groups than in the control group. Significant increase (*p* < 0.05) in the relative weight of the heart in rats treated with 1000 mg/Kg b.w. and equally significant increase (*p* < 0.05) of the relative weight of the kidney for the rats treated with 500 and 1000 mg/Kg b.w. could be correlated with a possible toxic effect of the plant at these doses since the kidney is one of the main organs involved in the elimination of xenobiotics in the body [24], but the histological sections of the kidney observation did not reveal any morphological alteration of this organ so this hypothesis is thus rejected.

The liver and kidneys are targeted organs for chemicals (drugs and poisons) because of their role in the detoxification and excretion processes of the body. These organs are extremely useful for toxicity studies because of their sensitivity to harmful compounds and their potential to predict possible toxicity. Changes in the weight of these vital organs related to toxicity are frequently accompanied by histopathological modifications. However, changes in the weight of certain organs such as the lungs, heart and spleen have less impact on toxicity because of their limited role in eliminating harmful substances from the body [25, 26].

Histopathological examinations performed on liver and kidney of treated and controls rats showed a normal kidney architecture and a liver with preserved hepatic architecture. The slight degeneration observed at the highest dose of ICR as compared to the control could be due to the toxic effect of this plant since drug/chemical-mediated hepatic injury is the most common manifestation of drug toxicity [27].

The analysis of serum and urinary biochemical parameters during toxicity studies provides important information on the functioning of specific tissues of the body such as the liver and kidneys that are highly involved in the metabolism and excretion of plant extracts that are mixtures of chemicals [28]. Apart from Aspartate aminotransferase and urinary creatinine, we did not find any other significant variations of the other biochemical parameters in the ICR treated groups compared to the control groups.

A significant decrease (*p* < 0.05) of aspartate aminotransferase levels we observed in all male and female animals treated with ICR at different doses (250, 500 and 1000 mg/Kg b.w.). Similar results were obtained by Chunlaratthanaphorn [23], who showed the ability of the aqueous extract of *Imperata cylindrica* to induce a decrease in AST levels at a dose 1200 mg/Kg b.w. Regarding ALT concentrations, we did not noted any significant variations within all group tests, and based on these results, ICR would not affect the proper functioning of the liver because following acute hepatocellular injury, there will be moderate to marked increase in both serum ALT and AST even though ALT is more reliable to detect for both acute and sub-acute hepatocellular injuries [29]. The serum hepatic leakage enzymes include alanine aminotransferase (ALT), aspartate aminotransferase (AST), sorbital dehydrogenase and glutamate dehydrogenase. Following hepatocellular injury or alterations in liver membrane permeability, these enzymes leak out of the membrane into peripheral blood. However, for practical purposes ALT and AST are currently widely used to assess liver functions [30].

Creatinine is a waste product that is produced by the muscles through normal contraction. Serum creatinine levels are constant and proportional to the muscle mass. Creatinine is excreted from the body through the kidneys. Then creatinine provides a good measure of how well the kidneys are working. In this study, we noted a significant decrease (*p* < 0.05) in urinary creatinine concentration in females and males treated with ICR at 500 mg/kg b.w. A low urine creatinine level could indicate reduced kidney function, but however, a factor like improper urine collection may also affect urine creatinine level [31]. With the exception of a significant decrease in LDL (low density lipoprotein) in the group of animals treated with ICR at 250 mg/Kg b.w., a slight non-significant increase of the total cholesterol (TC) and triglyceride (TG) was observed in groups of animals treated with 250 mg/kg, all other groups of animals treated with ICR showed a normal lipid profile compared to the control group. LDL cholesterol (low density lipoprotein) is a fat essential for the proper functioning of the body. LDL is also known as bad cholesterol because its increase is linked to an increase in cardiovascular risk. The decrease of this particular lipid level by ICR confirms the results of the work of Mak-Mensah et al. [32], Rouslin et al. [33] and Sulistyowati et al. [34] which showed that the plant used in this study is effective against a cardiovascular disorder such as hypertension, since it is widely accepted that hypertension is associated with an increased blood levels of low-density lipoprotein (LDL).

To determine the intravascular effect and bone marrow activity in rats treated with the extract, hematological parameters of female and male rats were examined as presented in Tables 7 and 8. Males treated with ICR at 500 and 1000 mg/Kg b.w. showed a significant increase (*p* < 0.05) of the levels of lymphocyte and MCHC; we had also noticed that females treated with the same doses of ICR showed a significant increase (*p* < 0.05) in Platelets levels compared to the control groups. In all groups of females treated with ICR, there was a significant decrease (p < 0.05) in granulocyte levels; while in males, there was a significant increase (*p* < 0.05) in granulocyte levels compared to the control groups. Several toxicological studies had shown that plant extracts can affect certain hematological parameters as it has been observed in this work [23, 35, 36].

Results obtained in this study may help for further animal studies using ICR*.* In effect, the leaf extract of this plant also displayed cytotoxic effects against cancer cells lines [9], though in-depth studies were not done as for ICR. The fact that the present investigation did not consider leaf extract and water extraction are limitations of this study. In effect, for a given plant, if both the leaves and roots present approximatively similar pharmacological activities like the case of *Imperata cylindrica* for their anticancer activities*,* for a sustainable production it is advisable to use leaves as source of drug since this would avoid the destruction of the whole plant, and moreover leaves are much more easier to get in large amount compare to roots. Hence further investigations of the toxicity of the leaf extract of *Imperata cylindrica* will also be performed. Nonetheless, the rationale of selecting the roots of *Imperata cylindrica* in the present work comes to the fact they are abundantly used as spices, commercially available in African Market.

## Conclusion

The acute toxicity results of ICR indicated an LD_50_ greater than the 5000 mg/Kg b.w. dose, suggesting that root methanol extract of this plant is non-toxic and could be used safely at single administration. However, prolonged oral administration of the methanol extract of ICR at 250 and 500 mg/Kg b.w. can lead to the variation of some biochemical and hematological parameters without causing damage of vital organs involved in the metabolism and excretion of xenobiotics from the body. At 1000 mg/Kg b.w., ICR can cause slight liver damage. Therefore, safety measures should be taken before oral ingestion of ICR for therapeutic purposes or for other uses, and prolonged use should be discouraged at high doses.

## Supplementary information

**Additional file 1.**

## Data Availability

All data generated or analyzed during this study are included in this published article.

## Abbreviations

ALT: alanine aminotransferase
AST: aspartate aminotransferase
b-w-: body weight
EDTA: ethylene diamine tetra-acetic acid
HCT: Hematocrit
Hb: hemoglobin
HDL: high density lipoprotein
ICR: methanol extract of the roots of *Imperata cylindrica*
MCHC: mean corpuscular hemoglobin concentration
MCV: mean corpuscular volume
MCH: mean corpuscular hemoglobin
LD_50_: medium lethal dose
LDL: low density lipoprotein
PLT: platelets
RBC: red blood cells
TG: triglyceride
TC: total cholesterol
