# Supplementary material for: A botanical from the antiproliferative Cameroonian spice, Imperata cylindrica is safe at lower doses, as demonstrated by oral acute and sub-chronic toxicity screenings
Source: BMC Complement Med Ther. 2020 Sep 10;20:273. doi: 10.1186/s12906-020-03064-6 (PMC7488045; doi:10.1186/s12906-020-03064-6)

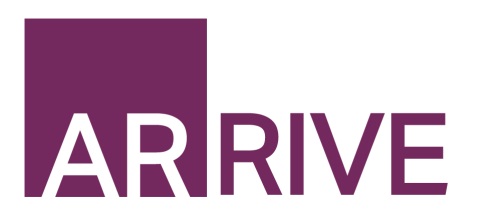


The ARRIVE Guidelines Checklist

Animal Research: Reporting In Vivo Experiments

Carol Kilkenny^1^, William J Browne^2^, Innes C Cuthill^3^, Michael Emerson^4^ and Douglas G Altman^5^

*^1^The National Centre for the Replacement, Refinement and Reduction of Animals in Research, London, UK, ^2^School of Veterinary Science, University of Bristol, Bristol, UK, ^3^School of Biological Sciences, University of Bristol, Bristol, UK, ^4^National Heart and Lung Institute, Imperial College London, UK, ^5^Centre for Statistics in Medicine, University of Oxford, Oxford, UK.*

|  | ITEM | RECOMMENDATION | Section/ Paragraph | |  |
| --- | --- | --- | --- | --- | --- |
| 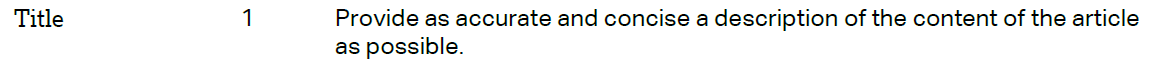 | | | | 1. Title | |
| 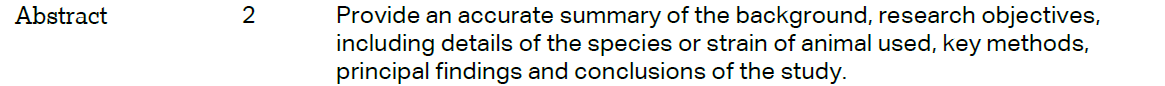 | | | | 2. Abstract | |
| INTRODUCTION | | | |  | |
| 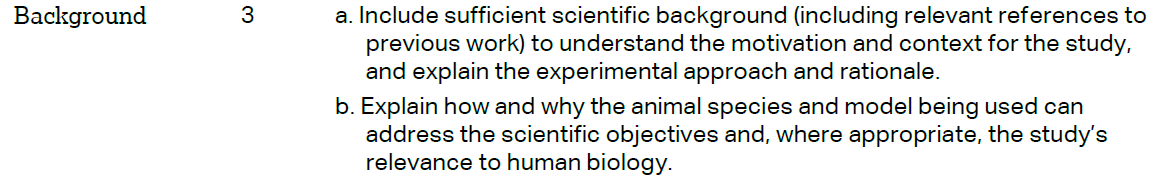 | | | | 3a. Background, Paragraph 2  3b.Background, Paragraph 3 | |
| 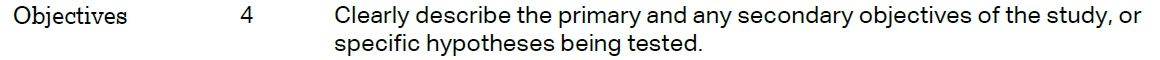 | | | | 4. Objective, Last paragraph | |
| METHODS | | | |  | |
| 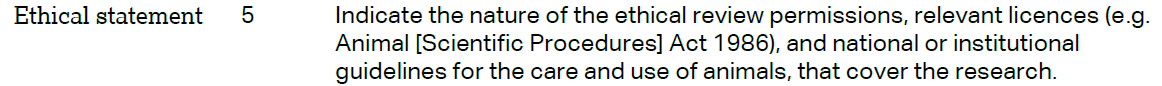 | | | | 5. Materials and Methods section, animals subsection | |
| 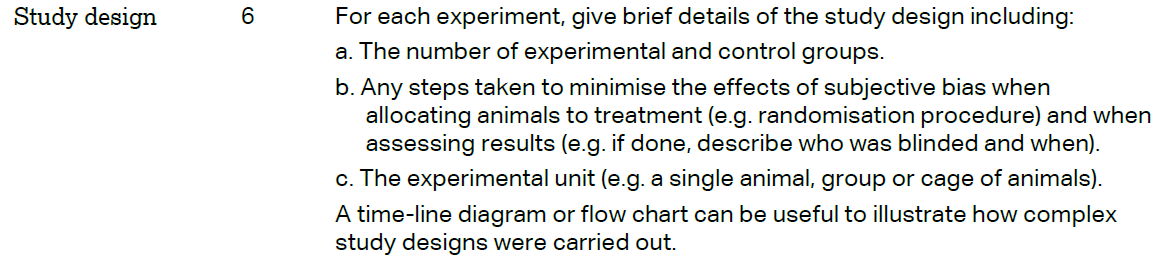 | | | | 6a. Materials and Methods section, animals subsection  6b. Materials and Methods section   - Acute oral toxicity study subsection - Sub-chronic oral toxicity study subsection - Collection of blood samples subsection - Assessment of biochemical parameters subsection - Assessment of haematological parameters subsection - Histology subsection   6c. Materials and methods section   - Acute oral toxicity study subsection (5 females rats for the test group and 5 females rats for the control groups) - Sub-chronic oral toxicity study subsection (4 groups of 10 animals per group : 5 females and 5 males). | |
| 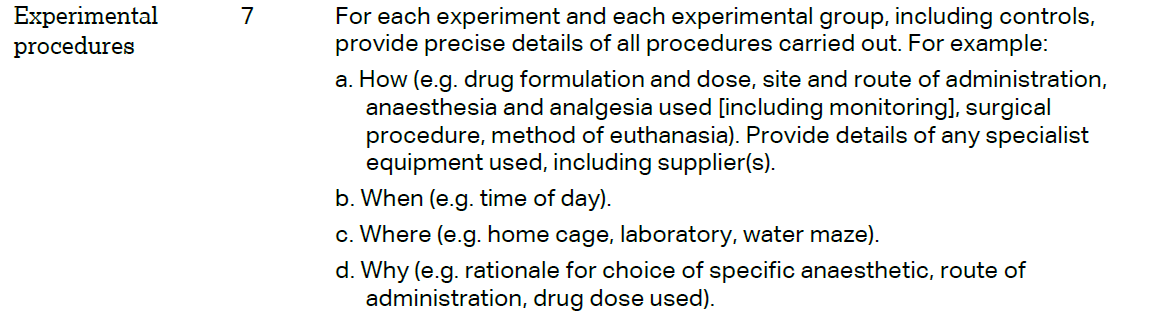 | | | | 7a. Materials and methods section   - Acute oral toxicity study subsection - Sub-chronic oral toxicity study subsection   7b. Materials and methods section   - Acute oral toxicity study subsection - Sub-chronic oral toxicity study subsection   7c. Materials and methods section, Animals subsection  7d. Materials and methods section   - Acute oral toxicity study subsection - Sub-chronic oral toxicity study subsection | |
| 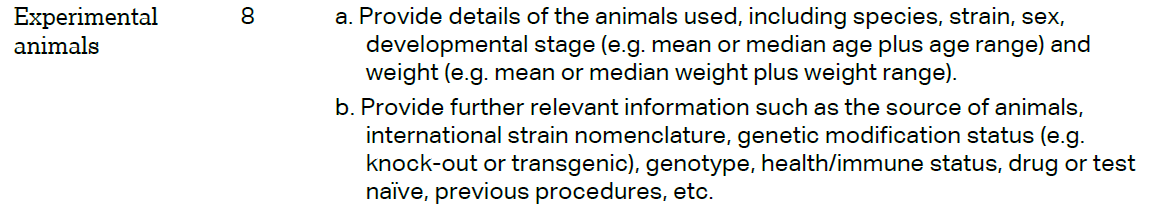 | | | | 8a. Materials and methods section   - Acute oral toxicity study subsection - Sub-chronic oral toxicity study subsection   8b. Materials and methods section, Acute oral toxicity study subsection (animals) | |

The ARRIVE guidelines. Originally published in *PLoS Biology*, June 2010^1^

| 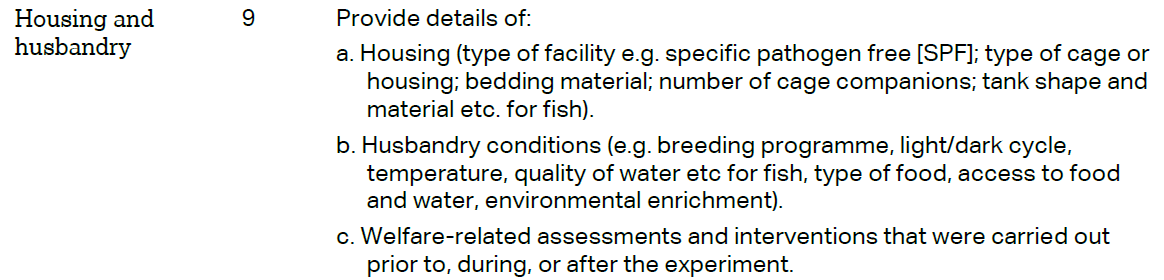 | 9a. Materials and methods section   - Acute oral toxicity study subsection - Sub-chronic oral toxicity study subsection   9b. Materials and methods section, Animals subsection  9c. Materials and Methods section : Acute oral toxicity study subsection and Sub-chronic oral toxicity study subsection | |
| --- | --- | --- |
| 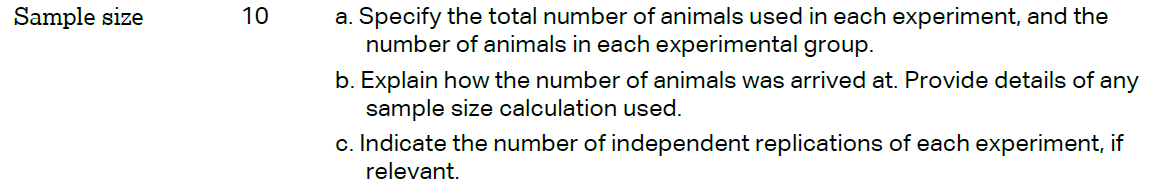 | 10a. Method section   - Materials and Methods section: Acute oral toxicity study subsection   and Sub-chronic oral toxicity study subsection  10b. Materials and Methods section  10c. Materials and Methods,   - Acute oral toxicity study subsection - Sub-chronic oral toxicity study subsection | |
| 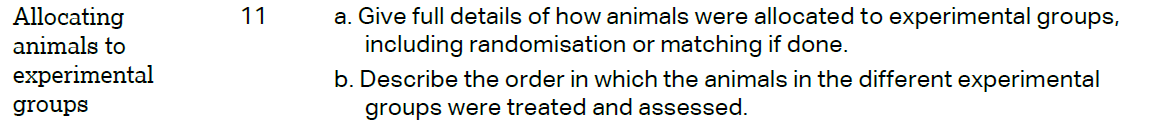 | 11a. Materials and Methods section   - Acute oral toxicity study subsection - Sub-chronic oral toxicity study subsection   11b. Materials and Methods section   - Acute oral toxicity study subsection - Sub-chronic oral toxicity study subsection | |
| 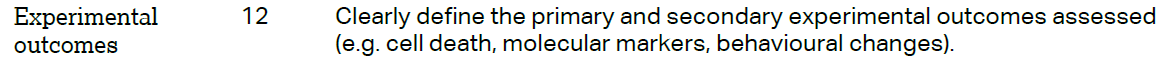 | 12. Materials and Methods section   - Acute oral toxicity study subsection - Sub-chronic oral toxicity study subsection - Collection of blood samples subsection - Assessment of biochemical parameters subsection - Histology subsection | |
| 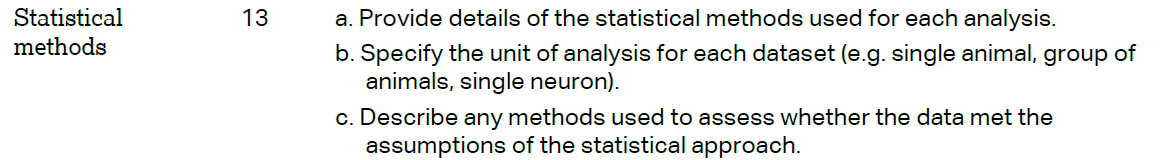 | 13a. Materials and Methods section : statistical analysis subsection  13b. Materials and Methods section : statistical analysis subsection  13c. Materials and Methods section: statistical analysis subsection | |
| RESULTS |  | |
| 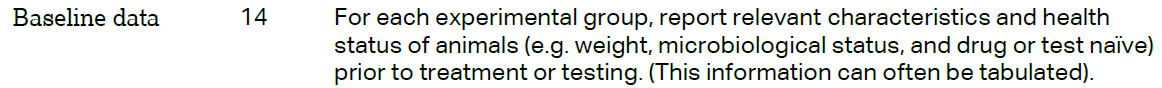 | 14. Results section, Tables 1-8 (dose in mg/kg) | |
| 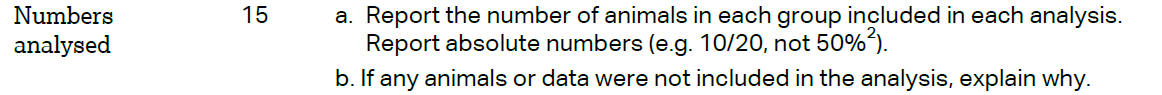 | 15a. Throughout results Section  15b. Not applicable | |
| 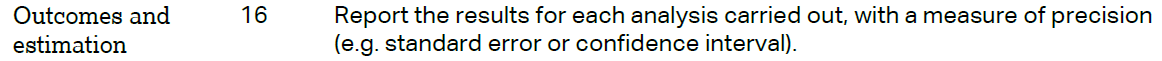 | 16. Results section, Tables 1-8 | |
| 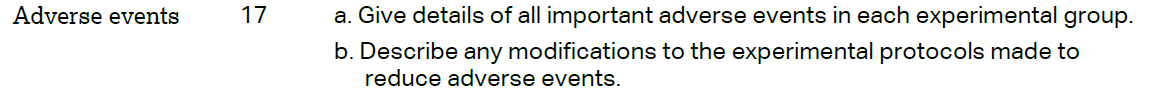 | 17. Not applicable | |
| DISCUSSION |  | |
| 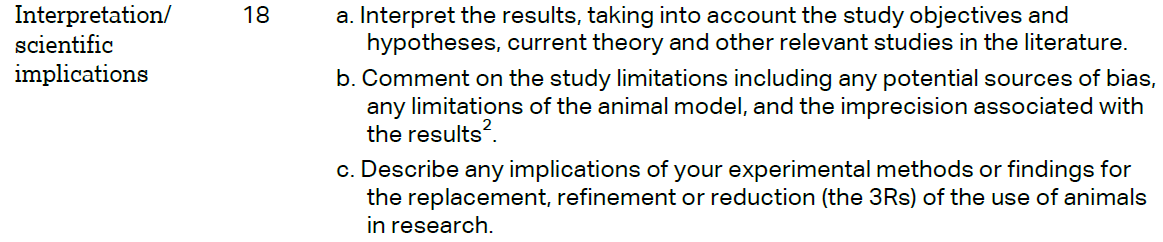 | 18. Throughout Discussion Section | |
| 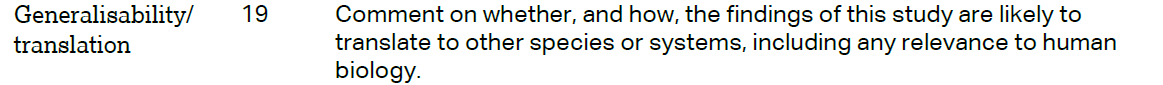 | 19. Not applicable | |
| 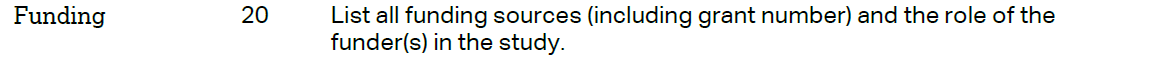 | | 20. No funding |


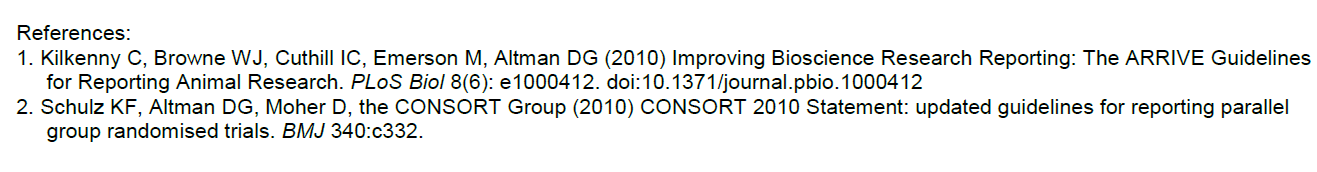

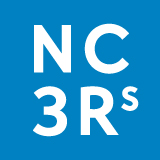

Supplement: Supplementary file 1 — Additional file 1. [file 12906_2020_3064_MOESM1_ESM.docx]
